# Supplementary material for: Prosthetic forefoot and heel stiffness across consecutive foot stiffness categories and sizes
Source: PLoS One. 2022 May 10;17(5):e0268136. doi: 10.1371/journal.pone.0268136 (PMC9089881; doi:10.1371/journal.pone.0268136)
Supplement: S1 Appendix — Testing was completed to evaluate variation in stiffness between “duplicate” prosthetic feet (i.e., feet ordered from the manufacturer at different times but identical in model, size, and stiffness category). Pairs of duplicate size 27 cm feet at two stiffness categories (i.e., corresponding to user weights of 68.0 and 90.7 kg (150 and 200 lb.)) of each foot type were tested. A total of 10 sets of duplicate feet were therefore compared. Testing was performed at pylon progression angles of -10° and +20° to simulate heel and forefoot loading, respectively. The experimental setup and all other procedures otherwise matched those described in the manuscript. Calculated linear stiffness was very similar (mean difference of 2.4 ± 1.3%) between all pairs of duplicate feet, for all foot types and stiffness categories (Fig A). The data between duplicate feet were similar both in calculated linear stiffness values and in the features observed in the force-displacement curves, suggesting repeatability in manufacturing across multiple samples of the same foot model. Similar results were observed in a previous study which tested multiple samples of a commercial foot [21]. (DOCX) [file pone.0268136.s001.docx]

**S1 Appendix. Testing of duplicate foot models**

Testing was completed to evaluate variation in stiffness between “duplicate” prosthetic feet (i.e., feet ordered from the manufacturer at different times but identical in model, size, and stiffness category). Pairs of duplicate size 27 cm feet at two stiffness categories (i.e., corresponding to user weights of 68.0 and 90.7 kg (150 and 200 lb.), respectively) of each foot type were tested. A total of 10 sets of duplicate feet were therefore compared. Testing was performed at pylon progression angles of -10° and +20° to simulate heel and forefoot loading, respectively. The experimental setup and all other procedures otherwise matched those described in the manuscript.

Calculated linear stiffness was very similar (mean difference of 2.4 ± 1.3%) between all pairs of duplicate feet, for all foot types and stiffness categories. The data between duplicate feet were similar both in measured linear stiffness values and in the features observed in the force-displacement curves (Fig A), suggesting repeatability in manufacturing across multiple samples of the same foot model. Similar results were observed in a previous study which tested multiple samples of a commercial foot [1] .

**Fig A. Force-displacement data for pairs of duplicate prosthetic foot models of the same size and stiffness category.**

Force-displacement data for pairs of duplicate prosthetic foot models for example 27cm commercial feet. Unloading portions of the forefoot curves are shown, while loading portions of the heel curves are shown. Data shows close alignment of duplicate prosthetic foot models.


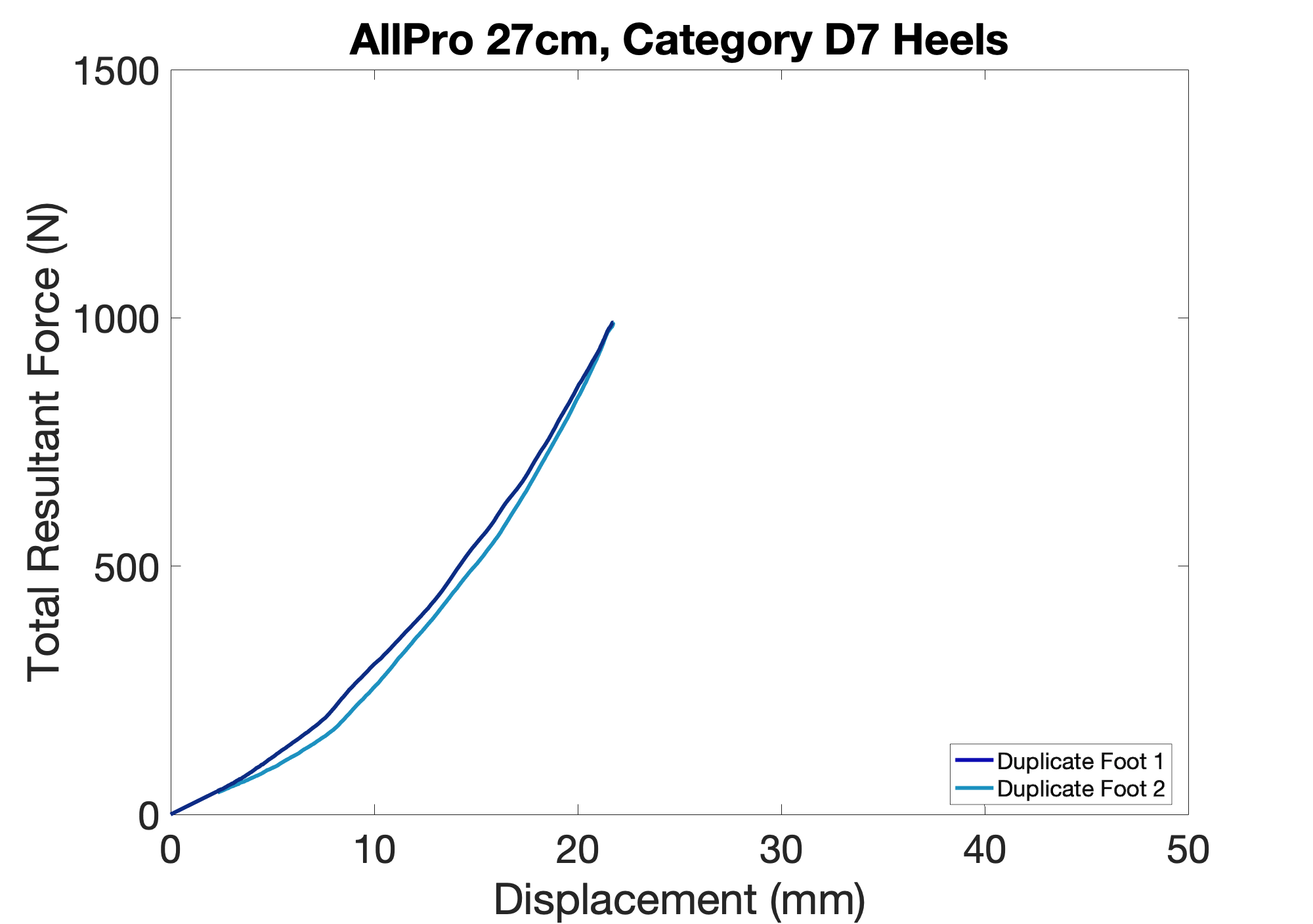

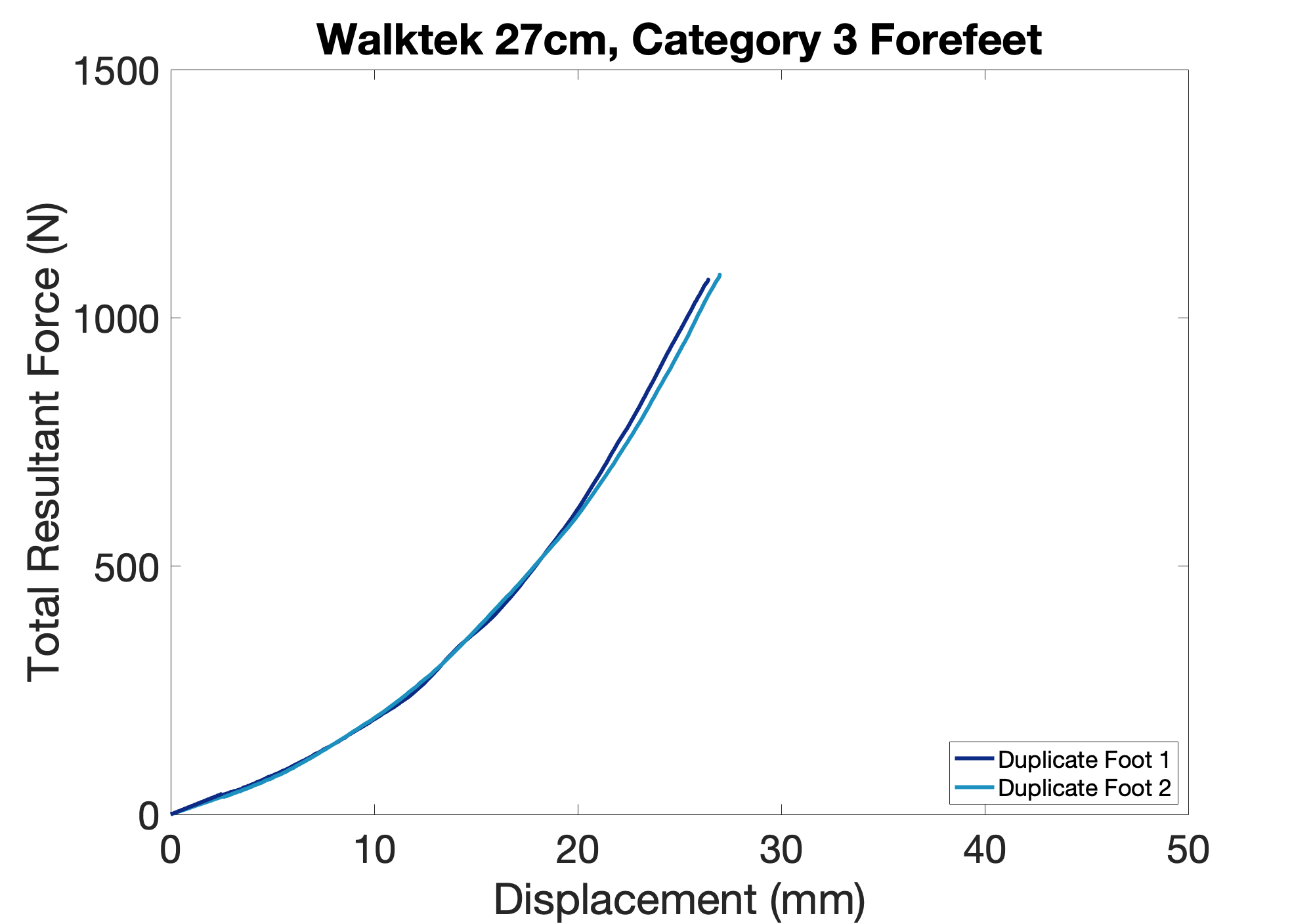
**
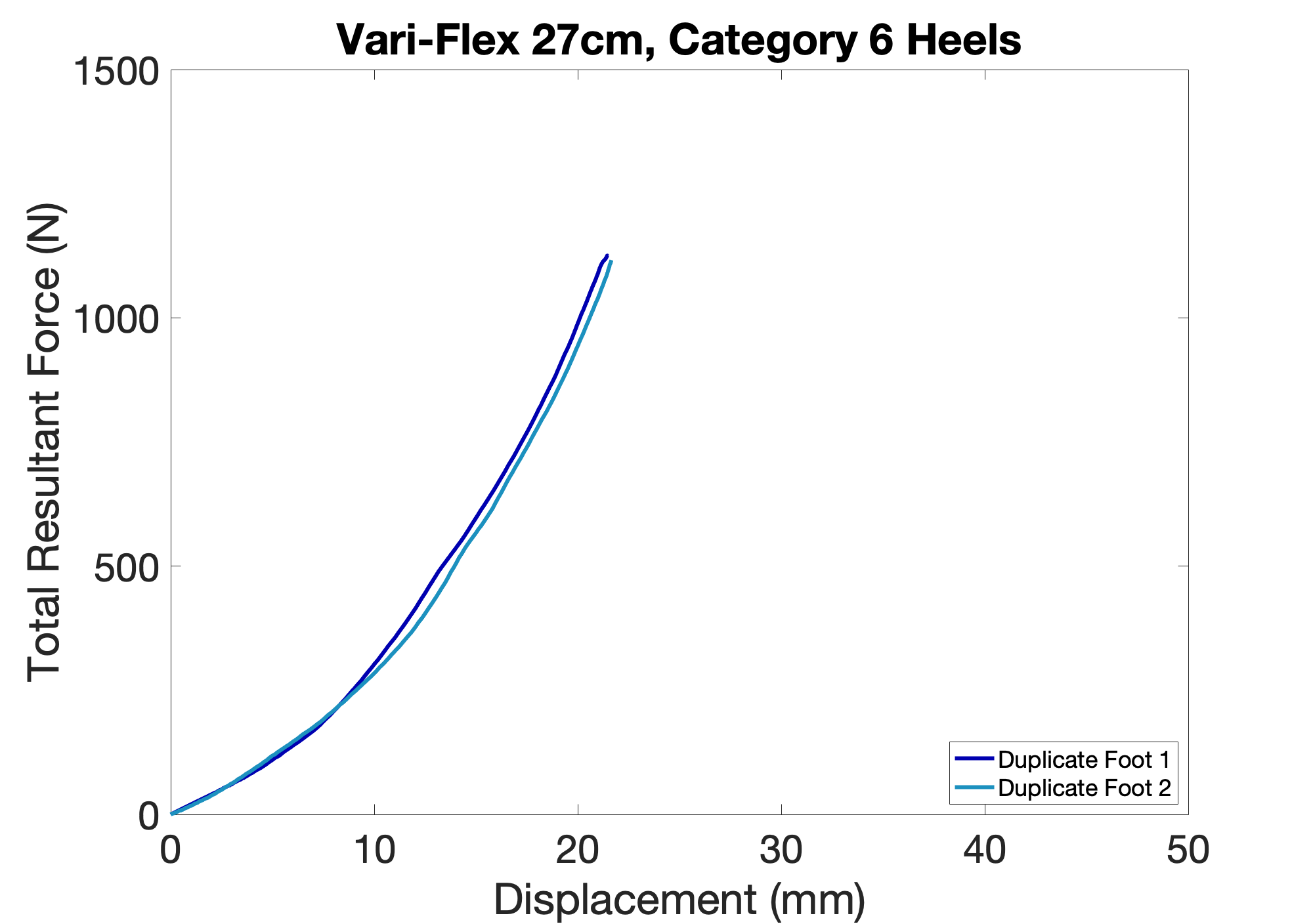
**

**References**

1. Geil MD. Energy loss and stiffness properties of dynamic elastic response prosthetic feet. JPO. 2001;13(3):70-3. PubMed PMID: 00008526-200109000-00011.
