# Supplementary material for: Prosthetic forefoot and heel stiffness across consecutive foot stiffness categories and sizes
Source: PLoS One. 2022 May 10;17(5):e0268136. doi: 10.1371/journal.pone.0268136 (PMC9089881; doi:10.1371/journal.pone.0268136)
Supplement: S2 Appendix — Example force-displacement data including highlighted areas representing the low and high-load areas of the curve used to determine relative difference in stiffness. Force-displacement curves also demonstrate method of linear stiffness calculation (i.e., linear stiffness used to quantify stiffness for each foot). (DOCX) [file pone.0268136.s002.docx]

**S2 Appendix. Calculation of linear stiffness and determination of relative difference between low-load stiffness to high-load stiffness**

Calculated linear stiffness was determined using linear regression models fit to two data points (i.e., the minimum point (50 N) and the mean target user body weight specified by each manufacturer for the respective foot stiffness category) on the force-displacement curve (Table 1). Stiffness was calculated using data from the unloading portions of the forefoot force-displacement curves and from the loading portions of the heel curves. The selected portions of the curves were chosen to reflect stiffness behavior representative of foot loading during the respective phase of gait (i.e., loading of the heel during early stance and unloading of the forefoot during late stance).

We used the following method to quantify the relative difference between low-load stiffness compared to high-load stiffness. Two measurements were used to establish a percentage of difference in stiffness behavior during low loads compared to during high loads: high-load stiffness and low-load stiffness. The high-loads region of the force-displacement data was defined as the load range within ± 5% of the expected peak ground reaction force for an average user (i.e., 10% region around the point representative of mean user body weight (Table 1)). This was intended to provide an estimate of linear stiffness at an instantaneous, effective body weight load range (i.e., the stiffness experienced by an average user during heel loading or forefoot unloading). The low-loads linear stiffness value was found using the same number of data points at low loading (i.e., an equivalent range of the force-displacement curve, starting from the minimum load). The relative difference between low-load and high-load stiffness was then calculated using the following formula:

|  | $\% Difference in Low vs High Load Stiffness = \frac{\sigma_{high}-\sigma_{low}}{\sigma_{high}}*100\%$ | (1) |
| --- | --- | --- |

A greater percentage difference indicates a larger magnitude of change between the stiffness experienced at low loads compared to that at high loads (i.e., greater nonlinearity).

**
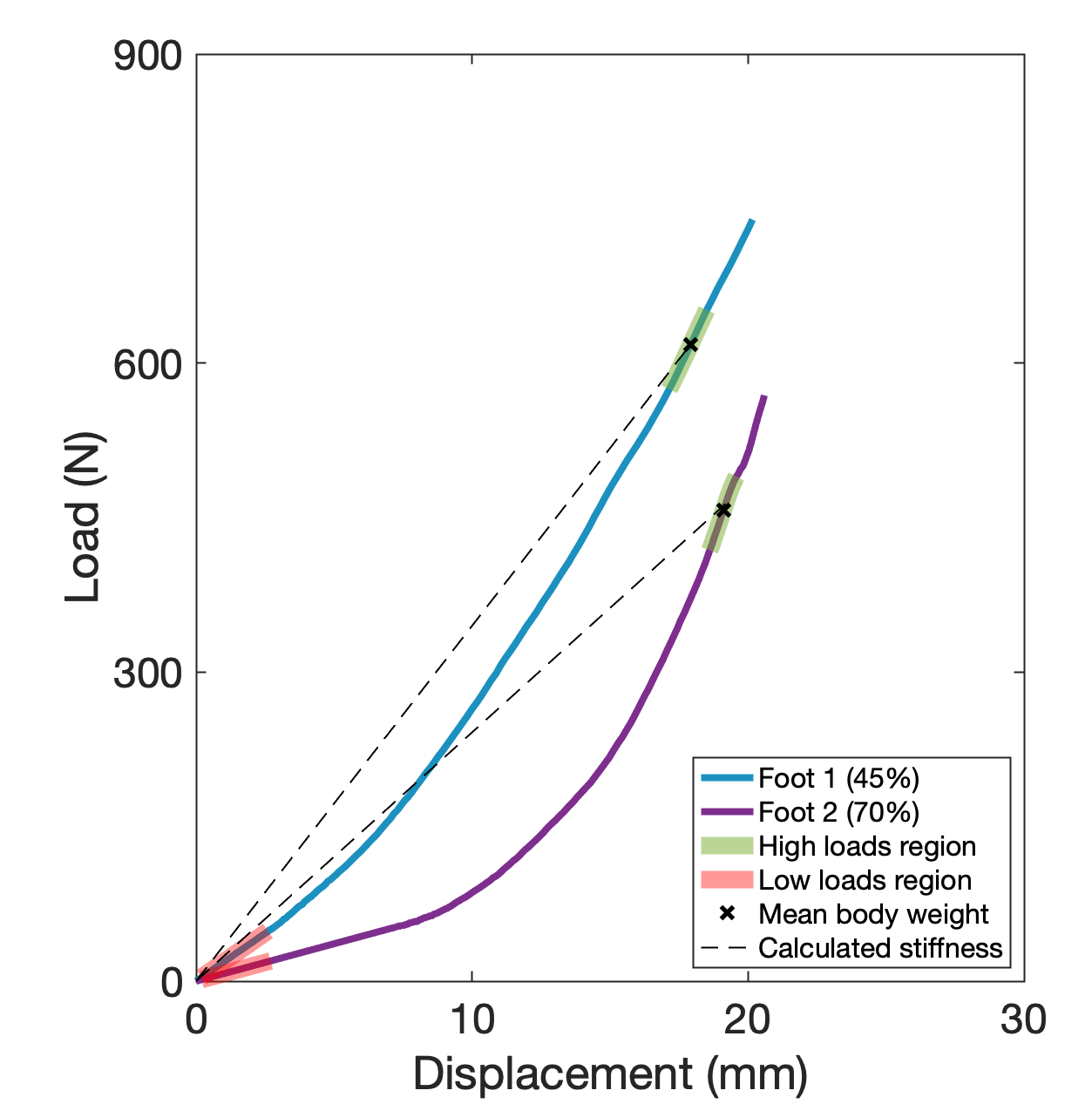
**

**Fig A. Example force-displacement data demonstrating methods used in determining stiffness and relative stiffness**

Example force-displacement data for two prosthetic feet: one foot with 45% low- to high-load relative stiffness and one foot with 70%. Calculated linear stiffness shown with dashed line from zero to mean body weight load. Areas of each curve highlighted to represent the low and high-load regions used to determine relative difference in stiffness.
