# Supplementary material for: Prosthetic forefoot and heel stiffness across consecutive foot stiffness categories and sizes
Source: PLoS One. 2022 May 10;17(5):e0268136. doi: 10.1371/journal.pone.0268136 (PMC9089881; doi:10.1371/journal.pone.0268136)
Supplement: S3 Appendix — (DOCX) [file pone.0268136.s003.docx]

**S3 Appendix. Statistical models used in linear mixed effects analyses**

Linear mixed effects regression was used to assess associations between calculated forefoot or heel stiffness (dependent variable) and stiffness category (independent fixed effect) with foot size as a fixed effect covariate, modeled as categorical. Foot type and foot type by stiffness category interaction were random effects. Stiffness category across feet was scaled so that a value of 1 represented minimum stiffness category (i.e., rated for 59.0 kg (130 lb.)) and 5 represented maximum category (i.e., rated for 113.4 kg (250 lb.)). Scaling was distributed equally within this range depending on the number of manufacturer stiffness categories for each foot type and size. Likelihood ratio tests were carried out to test the hypothesis of no association between calculated stiffness and stiffness category (i.e., the stiffness category coefficient is equal to zero) to test for variability in this association across foot types (by testing the significance of the foot type by stiffness category interaction term). To address our secondary hypothesis, the influence of foot size on the association between calculated stiffness and stiffness category was examined by testing the significance of stiffness category by size interaction terms. Thus, the linear mixed models were of the following forms:

|  | $Y_{ij}=\beta_{0}+\beta_{1}X+\gamma_{1}W_{1}+\gamma_{2}W_{2}+b_{0i}+b_{1i}X+e_{ij}$ | (1) |
| --- | --- | --- |

Where Y*_ij_* = Stiffness outcome for the *i*th foot and *j*th stiffness category measurement (*i*=1, …, 5 feet; *j*=1 up to 5 stiffness category measurements)

X = stiffness category (ranging from 1 to 5)

W_1_ = dummy-coded variable for foot size 28

W_2_ = dummy-coded variable for foot size 29

β_0_ = mean stiffness outcome at foot size 27, and stiffness category=0—not an interpretable parameter

β_1_ = mean change in stiffness outcome per one unit increase in stiffness category—the main effect of interest

γ_1_, γ_2_ = mean difference in outcome between foot size 28 and foot size 29 vs. foot size 27-not the focus of this analysis

b_0_*_i_* = the random effect for foot type *i*

b_1_*_i_* = the random effect for foot type *i* by stiffness category interaction

e*_ij_* = residual error

For the secondary hypothesis, the following interaction terms were added to the above model:

|  | $\delta_{1}XW_{1}+\delta_{2}XW_{2}$ | (2) |
| --- | --- | --- |

Where the two δ coefficients are tested jointly for significance to determine if there is a significant interaction between stiffness category and foot size.
