# Supplementary material for: Prosthetic forefoot and heel stiffness across consecutive foot stiffness categories and sizes
Source: PLoS One. 2022 May 10;17(5):e0268136. doi: 10.1371/journal.pone.0268136 (PMC9089881; doi:10.1371/journal.pone.0268136)

**S5 Appendix. Force-displacement curves for heels, arranged by prosthetic foot type and size.**

All tested stiffness categories within each prosthetic foot type and size are shown. Force and displacement data from the last three loading cycles were averaged (i.e., mean line shown for each foot) and standard deviation across cycles is demonstrated by shaded regions around each force-displacement curve. Loading portions of the heel curves are shown. Since a minimum force was maintained throughout testing, the data were linearly extrapolated to 0 N to provide an estimate for displacement at low loads. This was completed using a linear model fit to the initial ten force-displacement data points to estimate the earliest linear stiffness behavior, thus data shown below 50 N of load has been extrapolated.


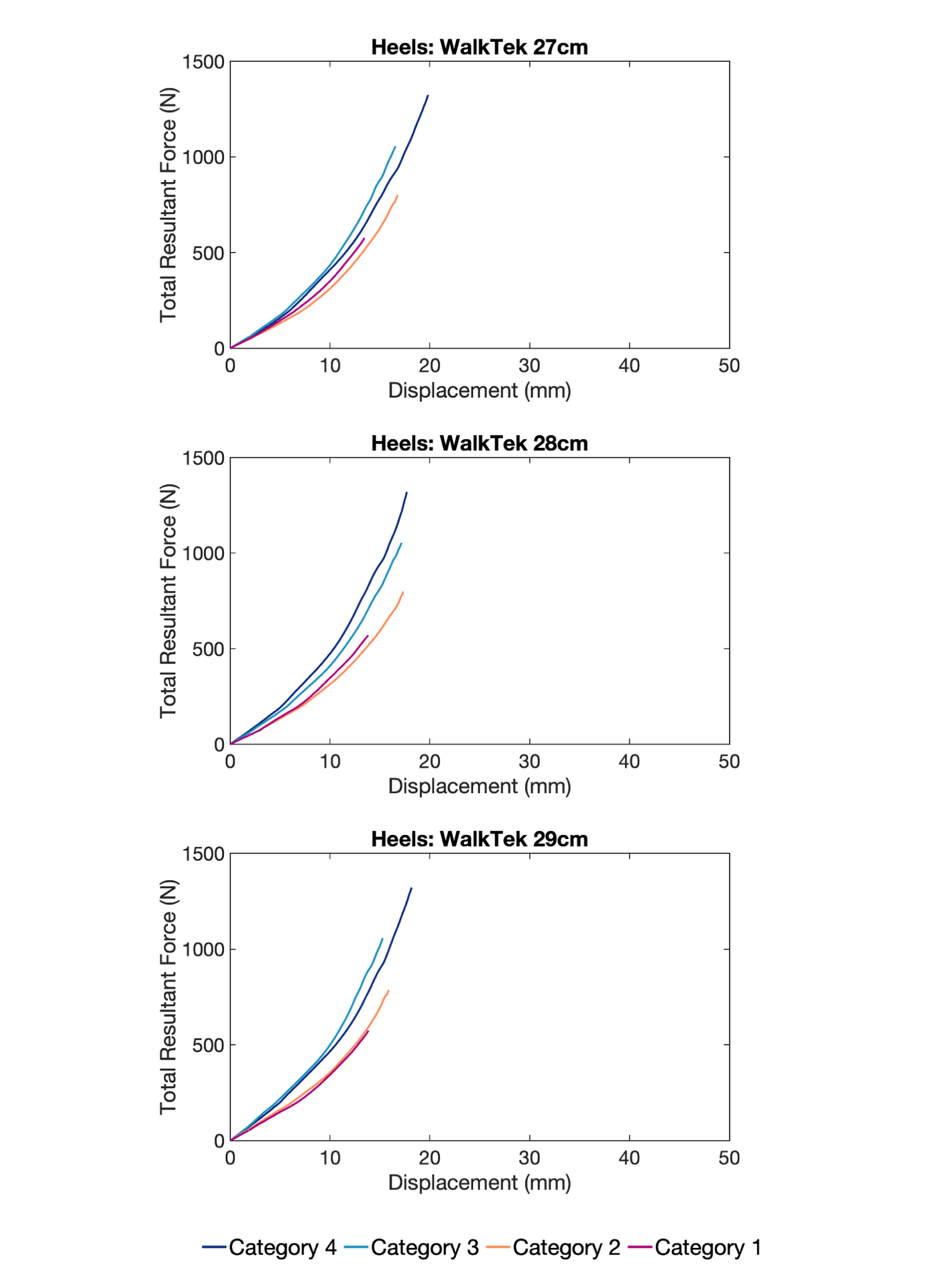


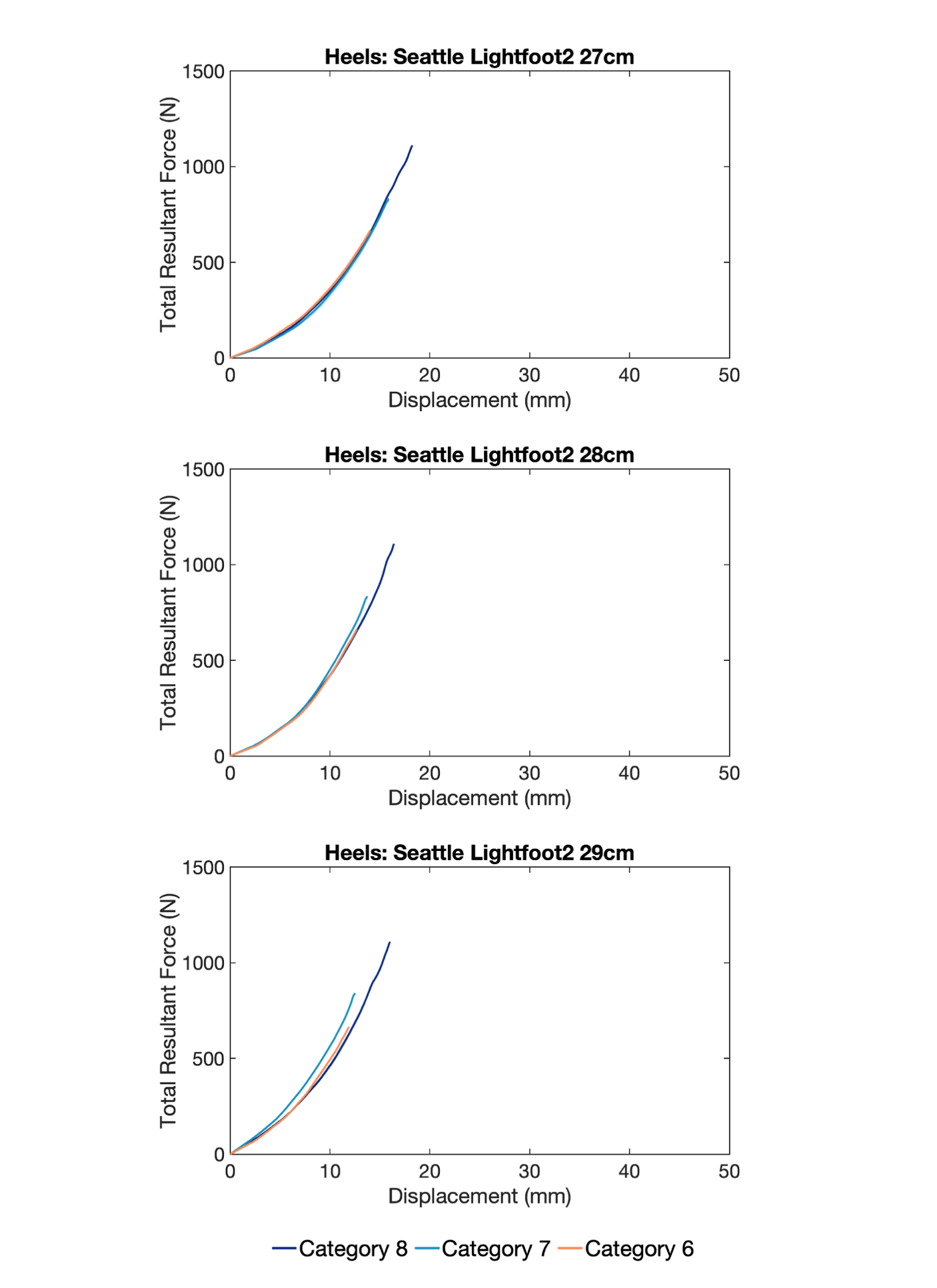


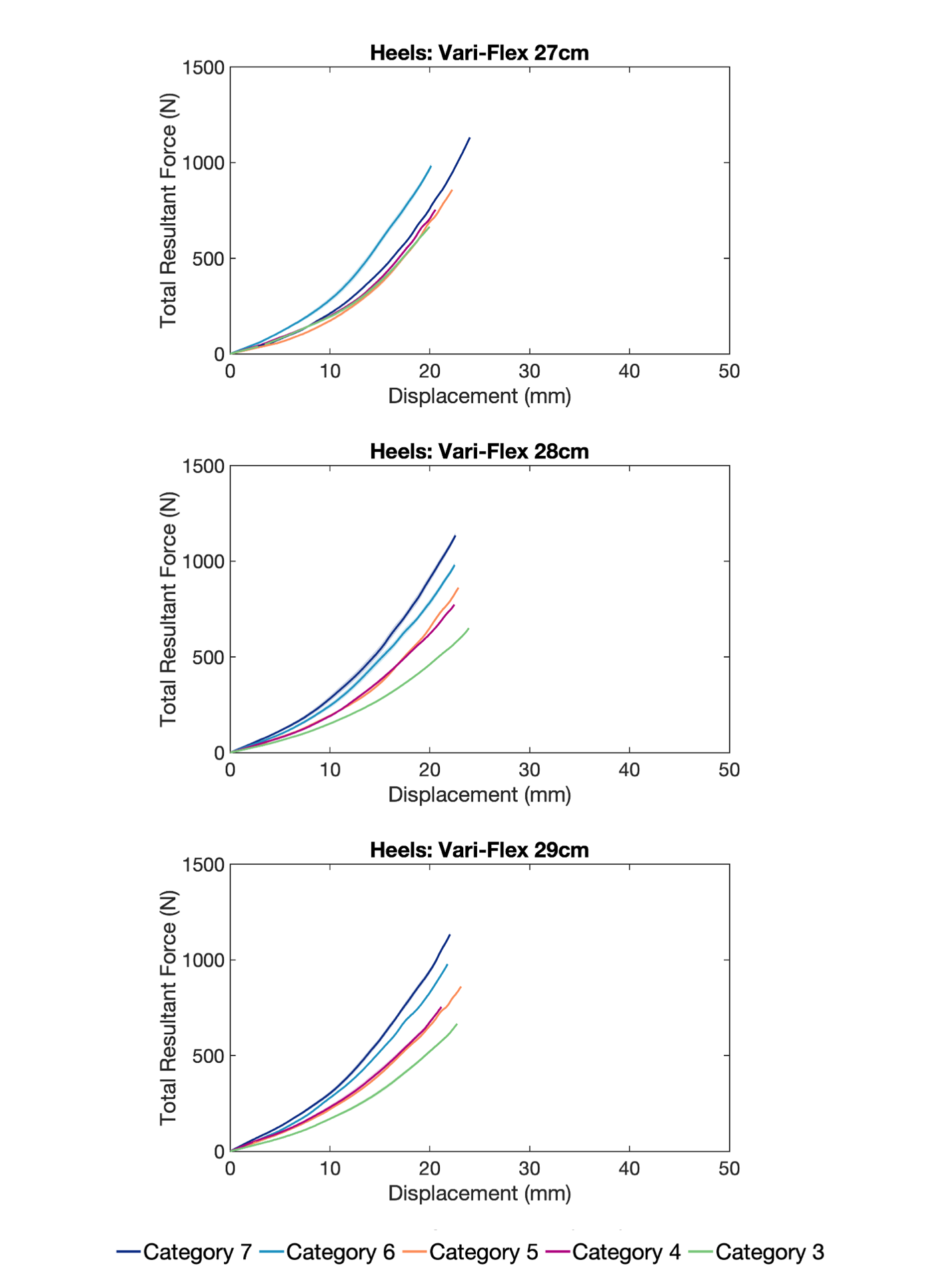


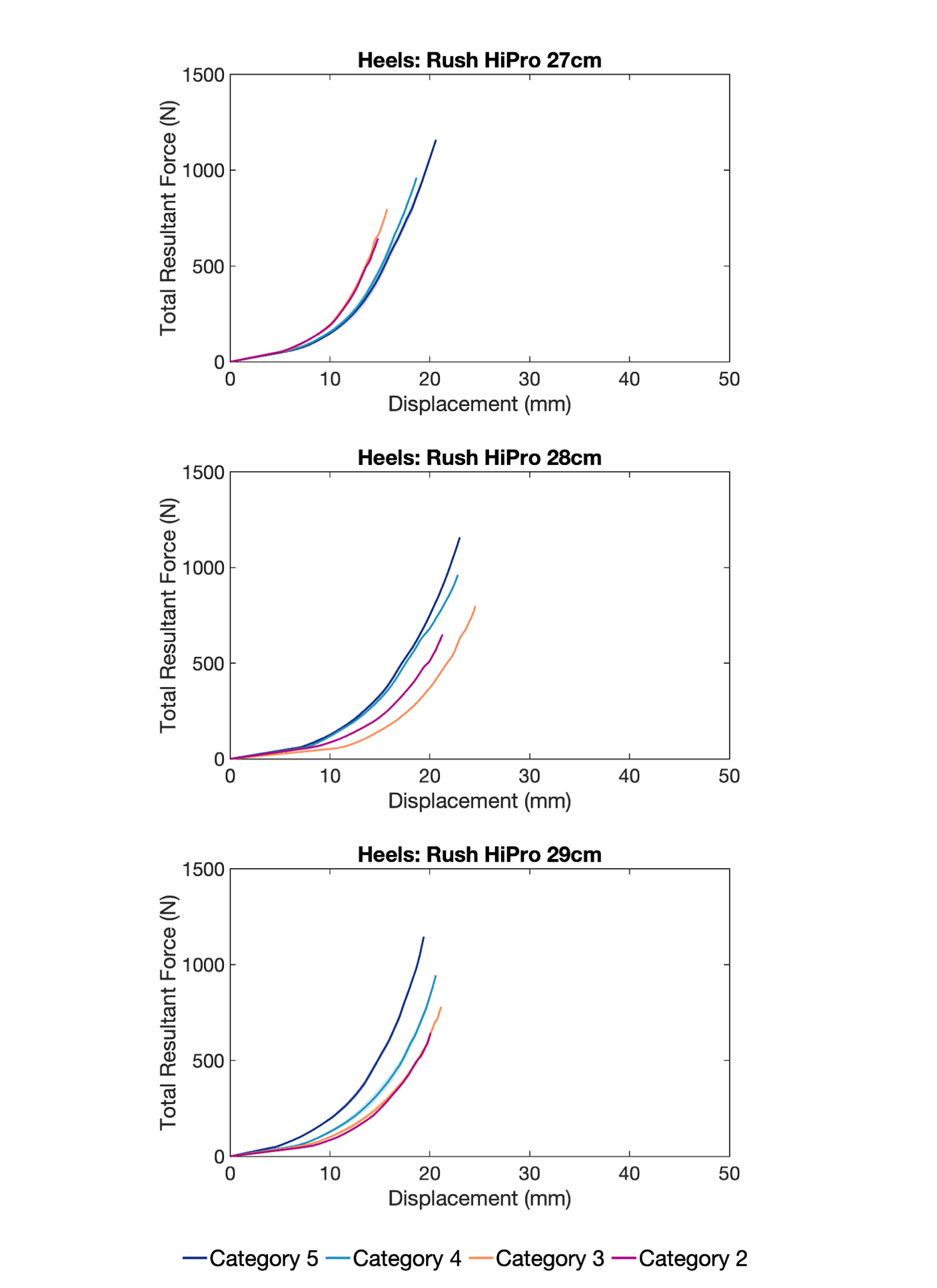


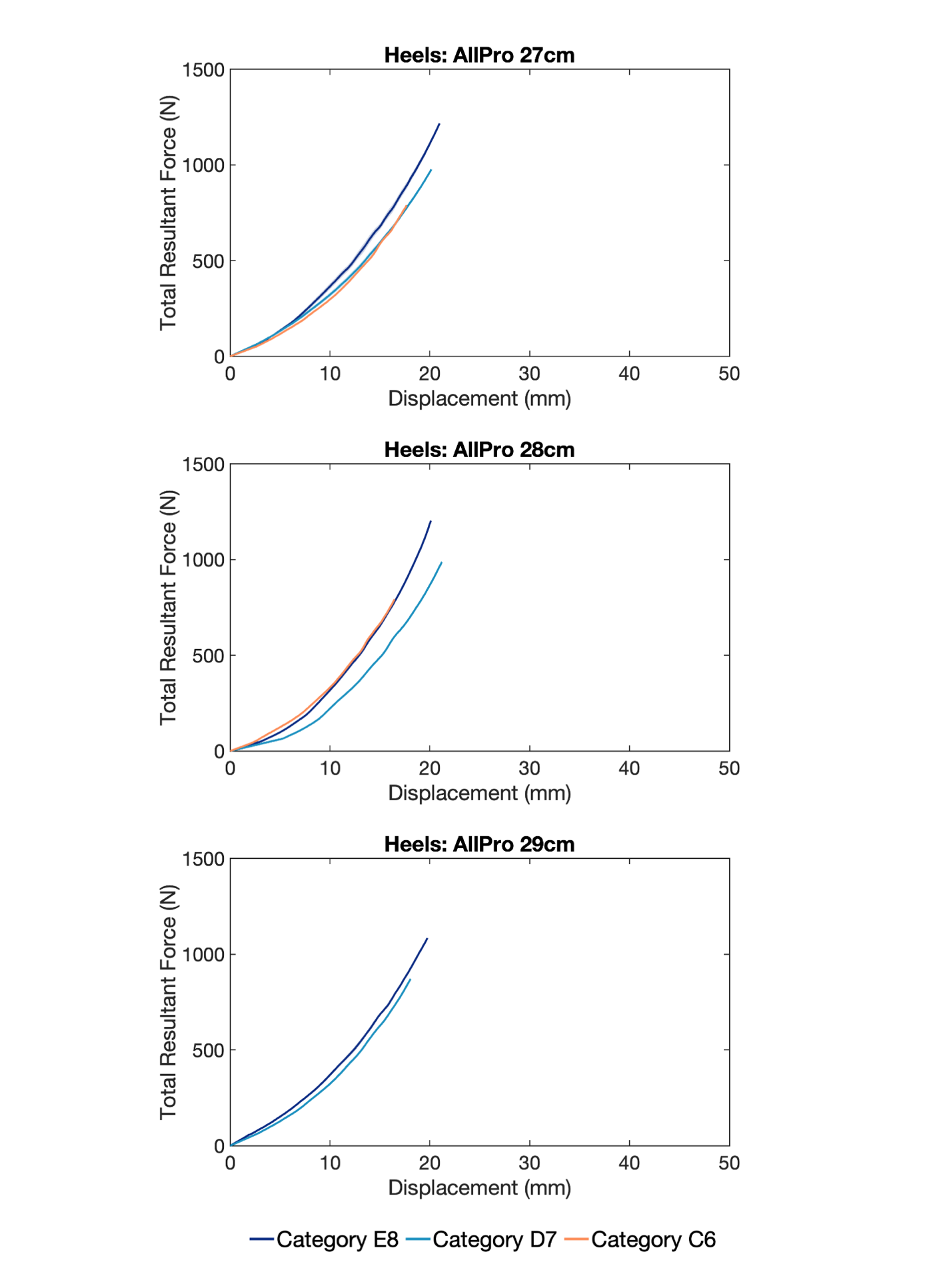

Supplement: S5 Appendix — All tested stiffness categories within each prosthetic foot type and size are shown. Force and displacement data from the last three loading cycles were averaged (i.e., mean line shown for each foot) and standard deviation across cycles is demonstrated by shaded regions around each force-displacement curve. Loading portions of the heel curves are shown. Since a minimum force was maintained throughout testing, the data were linearly extrapolated to 0 N to provide an estimate for displacement at low loads. This was completed using a linear model fit to the initial ten force-displacement data points to estimate the earliest linear stiffness behavior, thus data shown below 50 N of load has been extrapolated. (DOCX) [file pone.0268136.s005.docx]
