# Supplementary material for: Prosthetic forefoot and heel stiffness across consecutive foot stiffness categories and sizes
Source: PLoS One. 2022 May 10;17(5):e0268136. doi: 10.1371/journal.pone.0268136 (PMC9089881; doi:10.1371/journal.pone.0268136)
Supplement: S6 Appendix — (DOCX) [file pone.0268136.s006.docx]

**S6 Appendix. Forefoot and heel calculated stiffness comparison across studies for comparable prosthetic feet and pylon progression angles**

**Table A. Prosthetic Heel Stiffness Data Comparison (-15°)**

| **Previous Studies** | **Prosthetic Foot (Model, Size, Category)** | **Calculated Linear Stiffness (N/mm) from Current Study** | **Stiffness (N/mm) from "Previous Studies"** | **Difference in Calculated Stiffness (N/mm) Between Current Study and "Previous Studies"** |
| --- | --- | --- | --- | --- |
| Major et al., 2018,[1] | Seattle Lightfoot2, 27cm, Category 7 | 50.9 | 50.0 | 0.9 |
| Womac et al., 2019,[2] | Vari-Flex, 27cm, Category 4 | 35.6 | 40.1 | -4.5 |
|  | Vari-Flex, 27cm, Category 5 | 37.5 | 45.6 | -8.1 |
|  | Vari-Flex, 27cm, Category 6 | 47.4 | 51.9 | -4.5 |
|  | Vari-Flex, 27cm, Category 7 | 45.4 | 59.2 | -13.8 |
|  | Seattle Lightfoot2, 27cm, Category 6 | 55.0 | 55.8 | -0.8 |
|  | Seattle Lightfoot2, 27cm, Category 8 | 57.8 | 57.2 | 0.6 |

**Table B. Prosthetic Forefoot Stiffness Data Comparison (+20°)**

| **Previous Studies** | **Prosthetic Foot (Model, Size, Category)** | **Calculated Linear Stiffness (N/mm) from Current Study** | **Stiffness (N/mm) from "Previous Studies"** | **Difference in Calculated Stiffness (N/mm) Between Current Study and "Previous Studies"** |
| --- | --- | --- | --- | --- |
| Major et al., 2018,[1] | Seattle Lightfoot2, 27cm, Category 7 | 28.4 | 36.0 | -7.6 |
| Koehler-McNicholas et al., 2018,[3] | Vari-Flex, 27cm, Category 4 | 26.7 | 19.6* | 7.1 |
|  | Vari-Flex, 27cm, Category 6 | 33.6 | 25.7* | 7.9 |
|  | Rush HiPro, 27cm, Category 3 | 21.4 | 23.7* | -2.3 |
|  | Rush HiPro, 27cm, Category 4 | 27.9 | 33.4* | -5.5 |

*Estimated values based on force-displacement curves included in referenced article since this article did not include numerical stiffness values in the text or tables.

**References**

1. Major MJ, Scham J, Orendurff M. The effects of common footwear on stance-phase mechanical properties of the prosthetic foot-shoe system. Prosthet Orthot Int. 2018;42(2):198-207. doi: 10.1177/0309364617706749. PubMed PMID: 28486847.

2. Womac ND, Neptune RR, Klute GK. Stiffness and energy storage characteristics of energy storage and return prosthetic feet. Prosthet Orthot Int. 2019;43(3):266-75. doi: 10.1177/0309364618823127. PubMed PMID: 30688551.

3. Koehler-McNicholas SR, Nickel EA, Barrons K, Blaharski KE, Dellamano CA, Ray SF, et al. Mechanical and dynamic characterization of prosthetic feet for high activity users during weighted and unweighted walking. PloS one. 2018;13(9):e0202884. doi: 10.1371/journal.pone.0202884. PubMed PMID: 30208040; PubMed Central PMCID: PMCPMC6135372 organizations that could influence their work or pose conflicts of interest.
