# Supplementary material for: Prosthetic forefoot and heel stiffness across consecutive foot stiffness categories and sizes
Source: PLoS One. 2022 May 10;17(5):e0268136. doi: 10.1371/journal.pone.0268136 (PMC9089881; doi:10.1371/journal.pone.0268136)
Supplement: Attachment — Submitted filename: Response to Reviewers_2022_02_16.docx [file pone.0268136.s009.docx]

We would like to thank the editor and reviewers for their positive comments, and are glad that we have addressed the vast majority of questions and concerns from the previous round of edits. Our responses to the remaining questions from Reviewer 2 are shown below (Reviewer comments are pasted in italics, each followed by our response).

**Manuscript Number:** PONE-D-21-22884

**Title:** The Effect of Prosthetic Foot Stiffness on Foot-Ankle Biomechanics and Relative Foot Stiffness Perception in People with Transtibial Amputation

**Reviewer #2:**

1. *In the review responses, the authors wrote: “We used loading of the heel and unloading of the keel in the analysis to reflect the stiffness behavior representative of clinical use. Therefore, stiffness was based on foot loading or unloading during the respective phase of gait (i.e., heel loading during the heel contact phase and forefoot unloading during push-off).” [and in response to a different question from Reviewer 3] “Further, while feet are generally loaded (heel) and unloaded (keel) in the orientation we tested, they are not unloaded (heel) or loaded (keel) in that same orientation during walking.”*

*I am not following completely, but it seems that the argument has to do mainly with foot orientation? Though the foot orientation vs load are consistent in the material testing, it’s true they would be different during gait… with heel loading and keel unloading occurring more while the CoP is at the end of their respective cantilevers (I believe). But it’s still not clear to me why heel unloading and keel loading are less important than the heel loading and keel unloading... why aren’t these middle sections as “representative of clinical use?” They occur just as often, store/return similar amounts of energy, and have biomechanical implications… It seems it would be more intuitive to present only the loading phase of each, since we don’t know how much is lost due to hysteresis, making it an awkward comparison between heel and keel stiffnesses.*

Response:

We appreciate the reviewer giving us an opportunity to further clarify our methods and reasoning. In completing mechanical testing, we chose pylon progression angles (i.e., -15° and +20°) that were consistent with ISO standards 10328 and 22675. These pylon progression angles (i.e., shank angles) correspond to heel loading and forefoot unloading, respectively. These angles were also chosen to enable isolation of the heel and forefoot (keel) components, allowing for the determination of stiffness of each individual component.

As described in Jacqueline Perry’s seminal textbook, *Gait Analysis: Normal and Pathological Function*, during walking, initial contact (when prosthetic heel loading occurs) typically occurs between 0% and 2% of the gait cycle, corresponding with a shank angle range of approximately -14° to -16°. Therefore, a pylon progression angle of -15° is consistent with the heel loading phase of gait. The heel rocker ends at approximately 12% of the gait cycle, or at a shank angle of approximately 0°, when the entire foot comes in contact with the ground. Thus, physiologic heel unloading begins after a neutral shank angle (0°) and would not be represented at the -15° angle used in our testing. Similarly, terminal stance occurs between 31% and 50% of the gait cycle, which corresponds with a shank angle between 10° and 30°. During this phase of gait, the forefoot is loaded (as the ankle continues dorsiflexing) and then rapidly unloaded (following peak dorsiflexion). Peak dorsiflexion occurs at approximately 41 to 45% of the gait cycle, which corresponds to approximately 19-21° shank angle. Thus, physiologic forefoot loading would not be represented at the 20° angle used in our testing.

While we agree with the reviewer that stiffness quantification of the “middle sections” (i.e., where heel unloading and forefoot loading occur during physiologic gait) may also be important, in order to capture this information, mechanical testing would need to be completed at different pylon progression angles and would not isolate the forefoot and heel components. We therefore included the following wording in the limitations section to clarify for the reader. “Additionally, quasi-static loading performed at two, sagittal-plane pylon progression angles is not representative of loading throughout the full gait cycle, although it is consistent with previous studies.”

One reason that previous studies have included both loading and unloading data has been to quantify hysteresis as well as energy storage and return. However, neither of these were objectives of the present study.

Lastly, to address the reviewer’s final point, the comparisons of linear stiffness measurements made in the manuscript were within heels and keels, respectively. This study did not aim to compare heel versus forefoot stiffness.

Reference:

Perry, Jacquelin, and Judith M. Burnfield. "Gait analysis: Normal and Pathological Function 2nd ed." *California: Slack* (2010).

2. *[Previously, I asked:] Why is foot size a categorical variable? It is a measurable quantity that is definable across models, and you will lose power by treating it as a categorical variable. [Response:] While we appreciate this point, we respectfully disagree. We do not consider foot size to be a continuous variable because it contains a finite number of categories. In contrast, if foot size had infinite number of values between any two of the categories, then it may have been appropriate to consider it a continuous variable and treat it as such in our analysis. But the point isn’t that it must be “continuous” (as opposed to “discrete”)… only that it must be “numerical” (as opposed to “categorical.”) It is okay that it can only take a finite number of possible values, such as the few integer foot lengths… it is still numerical—and it has ordering and spacing properties that will add substantial value to the statistical model (and will thus be more correct). Since the statistics are an important part of this paper, I must push on this a bit.*

Response:

We agree with the reviewer that foot size is a numeric variable (more precisely, an integer variable with ordered levels – much like shoe size). However, this does not preclude modeling foot size as categorical. We justify our decision by presenting our model (i.e., foot size modeled as categorical) compared to the reviewer’s suggested change to the model (i.e., foot size modeled as numerical) of calculated stiffness in a bit more detail and by discussing the implications below.

Here are the estimated fixed effects when modeling size as numerical, as the reviewer suggested (StfCat is the foot stiffness category variable, Size..cm. is the size variable):

```
Fixed effects:
            Estimate Std. Error t value
(Intercept) 16.72230   17.26553   0.969
StfCat       3.83384    0.33007  11.615
Size..cm.    0.04186    0.61216   0.068
```

In contrast, here are the estimated fixed effects used in our model, where foot size is modeled as categorical (constructed using two dummy variables with size 27 as the reference category):

```
Fixed effects:
            Estimate Std. Error t value
(Intercept) 16.95560    2.02977   8.353
StfCat       3.81464    0.30683  12.432
Size28       2.87000    1.12050   2.561
Size29       0.01737    1.13810   0.015
```

The numerical size variable estimates a slope of change close to zero (i.e., 0.04). However, when the size variable is modeled as categorical, it shows that size 28 has a mean stiffness 2.9 N/mm greater than that for size 27, and is significant at *p* < .05 (as shown by the *t*-value). This also affects the slope term for the stiffness category variable, which is estimated with more precision when size is modeled as categorical (as demonstrated by the higher *t*-value in the 2^nd^ model).

Below is a comparison of the goodness of fit statistics for model 1 (foot size as continuous) vs. model 2 (foot size as categorical):

```
        npar    AIC    BIC  logLik deviance  Chisq Df Pr(>Chisq)
Model 1    5 324.76 334.89 -157.38   314.76
Model 2    6 318.40 330.55 -153.20   306.40 8.3641  1   0.003827 **
```

Model 2 demonstrates a lower AIC and BIC, with the likelihood ratio test favoring Model 2 compared to Model 1 (*p* = .0038). This finding supports our decision to model foot size as categorical. The reason for the improved precision of Model 2 compared to Model 1 using a numerical variable is likely due to the non-linear relationship between foot size and foot stiffness that was observed in these data (i.e., size 28cm had increased stiffness relative to both size 27 and 29cm).

We did not want to make any a prior assumptions as to the association between foot size and calculated stiffness (e.g., whether a linear relationship existed), and with only three size values (i.e., 27, 28, and 29cm), we could not model a nonlinear association with size as numerical. If we had more foot sizes, then modeling size as a numerical variable would potentially result in an improved model fit by adding polynomial or spline terms. However, given that we included three foot-sizes in this study and the model comparisons we presented above, we believe that the model we used is stronger.
